# Diversity Arrays Technology-based PCR markers for marker assisted selection of aluminum tolerance in triticale (*x Triticosecale* Wittmack)

**DOI:** 10.1007/s11032-015-0400-8

**Published:** 2015-11-03

**Authors:** Agnieszka Niedziela, Dariusz Mańkowski, Piotr T. Bednarek

**Affiliations:** Plant Breeding and Acclimatization Institute - National Research Institute, Radzików, 05-870 Błonie, Poland

**Keywords:** Triticale, Aluminum tolerance, Marker conversion, DArT

## Abstract

The tolerance of triticale (*x Triticosecale* Wittmack) cultivars to aluminum (Al) stress observed in acid soils is an important agronomic trait affecting seed yield. Traditionally, breeding of Al-tolerant cultivars was selection based; for example, using a physiological test. However, such selection methods are relatively slow and require numerous plants for phenotype evaluation. Alternatively, DNA-based molecular marker systems could be applied to identify markers useful for selection purposes. Among many marker platforms available, Diversity Arrays Technology (DArT) is one of the most promising. DArT markers preselected for conversion to specific PCR assays were chosen based on association mapping studies using diverse materials. Forty-nine DArT markers were selected and tested for redundancy based on their segregation patterns and sequences, and 40 were successfully converted into specific PCR assays. However, only 24 of these proved to be polymorphic. Where possible, the chromosomal locations of the converted markers were verified. The markers assigned to chromosome 7R that were the most highly correlated with Al-tolerant and non-tolerant plants were chosen for marker assisted selection using genetically diverse triticale materials.

**Electronic supplementary material:**

The online version of this article (doi:10.1007/s11032-015-0400-8) contains supplementary material, which is available to authorized users.

## Introduction

Triticale is a modern wheat and rye hybrid crop containing parts of the genomes of both species (Qualset and Guedes-Pinto 1996), which together determine its tolerance to aluminum ions (Al^3+^) present in a soluble form in acidic soils (Foy 1992). Aluminum (Al) toxicity affects plant growth and influences grain yield (Foy 1992; Kochian 1995). The majority of known internal and external mechanisms of Al tolerance in plants are associated with the exudation of organic acids and the chelating activity of these acids (Kochian 1995).

Studies in triticale indicate that Al tolerance is encoded by at least four loci, mapped to chromosomes 3R, 4R, 6R, and 7R (Ma et al. 2000; Budzianowski and Woś 2004; Niedziela et al. 2012, 2014). However, genetic mapping carried out in rye and wheat demonstrates that the trait is multigenic, with numerous genes dispersed across chromosomes including 3R, 4R, 6R, and 7R in rye (Anioł and Gustafson 1984; Fontecha et al. 2007; Silva-Navas et al. 2012) and 2A, 5A, 6A, 7A, 3B, 4B, 2D, 3D, 4D, and 7D in wheat (Anioł and Gustafson 1984; Anioł 1990; Cai et al. 2008; Raman et al. 2005; Ma et al. 2005).

Previous studies on triticale have not focused on the putative function of genes encoding Al tolerance, although it was suggested that the Al-activated malate transporter (ALMT) gene, present on chromosome 7R, may contribute to the trait (Niedziela et al. 2014). However in rye, five loci encoding Al-tolerance genes have been described (Fontecha et al. 2007; Silva-Navas et al. 2012). Among these loci, two are located on chromosome 7R: Alt4, encoding *Secale cereale* ALMT (ScALMT1), is responsible for malate exudation (Fontecha et al. 2007), whereas the Alt5 locus encodes *Secale cereale* Al-activated citrate transporter 1 (ScAACT1), which is involved in citrate exudation (Silva-Navas et al. 2012). There are also data indicating that the Alt2 locus on chromosome 3R encodes a putative STOP1 transcription factor that may regulate the expression of genes at the Alt4 and Alt5 loci (Silva-Navas et al. 2012). In wheat, genes affecting Al tolerance include malate and citrate organic acids, which are encoded by loci on chromosomes 4DL (Sasaki et al. 2004) and 4BL (Ryan et al. 2009), while the TaSTOP1 transcription factor maps to chromosome 3BL (Garcia-Oliveira et al. 2013).

In triticale, Al tolerance is largely defined by the action of the ALMT gene, which accounts for 36 % of phenotypic variance (Niedziela et al. 2014), whereas in rye 60 % of the variation in this trait (Silva-Navas et al. 2012) is explained by a QTL located on chromosome 7R (Fontecha et al. 2007), which may also correspond to the ALMT gene. In wheat, the genes located on chromosomes 4DL, 4BL, and 3BL are reported to account for up to 56, 50, and 49 % of the variance, respectively (Zhou et al. 2007; Cai et al. 2008; Navakode et al. 2009; Ryan et al. 2009). In triticale, wheat and rye genomes appear to interact with one another; however, the rye genome appears to exert most influence over Al tolerance (Ma et al. 2000; Budzianowski and Woś 2004; Niedziela et al. 2012).

Isolation and characterization of the ALMT gene in rye and wheat (Sasaki et al. 2004; Fontecha et al. 2007) has enabled the evaluation of PCR-based markers potentially useful in marker assisted selection (MAS) programs. In rye, based on rice/rye synteny, rice-derived PCR-based markers flanking the Alt4 locus were developed (Miftahudin et al. 2004, 2005; Collins et al. 2008; Benito et al. 2010). Similar studies in wheat enabled the design of allele-specific PCR primers based on variation in exon four, intron three, and the promoter region of the TaALMT1 gene (Sasaki et al. 2004; Raman et al. 2005, 2006; Sasaki et al. 2006). ALMT1 sequence specific repeat (SSR) and cleaved amplified polymorphic sequence (CAPS) markers can distinguish Al-tolerant and Al-resistant cultivars; however, these methods can only distinguish between two alleles (ALMT1-1 and ALMT1-2) of this gene (Sasaki et al. 2004; Raman et al. 2006), whereas testing of almost 500 wheat germplasm accessions with these markers revealed the presence of 22 haplotypes and it is known that there are very few universal allele-specific PCR markers for plant selection, especially in polyploid species (Raman et al. 2008).

Analyses directed toward the evaluation of markers for Al tolerance in triticale began several years ago (Niedziela et al. 2012, 2014). Initially these involved mapping studies using either biparental mapping populations (Niedziela et al. 2014) or association mapping studies based on diverse inbred lines (Niedziela et al. 2012). Numerous Diversity Arrays Technology (DArT) markers associated with Al-tolerant genes on chromosomes 7R and 3R, as well as on chromosomes 4R and 6R, were assessed, but specific PCR assays for these were not developed.

Marker conversion to PCR-based assays is generally tedious and involves many intermediate steps (Xu et al. 2001; McNeil et al. 2011). For example, conversion of Amplified Fragment Length Polymorphism (AFLP) and/or Random Amplified Polymorphic DNA (RAPD) markers into CAPS requires cloning, sequencing, and primer evaluation, followed by validation. Moreover, because of the possibility of the presence of multiple sequences within a single band (Xu et al. 2001; Mechanda et al. 2004; McNeil et al. 2011) and the fact that polymorphisms may be related to restriction sites (AFLP) or present within the region of primer annealing (RAPD), successful development of PCR-based markers is relatively limited (Shan et al. 1999; Dussle et al. 2002). Importantly, techniques such as RAPD or AFLP can amplify several markers tightly linked to the trait of interest. If the conversion to specific PCR conditions is successful, only a limited number of assays exhibit proper segregation patterns (Xie et al. 2008; Lee et al. 2010; JinPing et al. 2009). However, the majority of these problems do not appear to apply to DArTs, as these are based on strictly defined sequences, are usually relatively long, and primer design for their amplification is simple (Eckstein et al. 2008; Shahin et al. 2009; Macko-Podgórni et al. 2014; Fiust et al. 2015). The available evidence indicates that converted DArTs usually maintain the segregation pattern of the original DArT marker (Eckstein et al. 2008; Shahin et al. 2009).

The primary motivation for this study was to use available information on DArT markers associated with loci on chromosomes 4R, 6R, and 7R to evaluate PCR-based molecular markers for Al-tolerance genes applicable to the broad genetic pool of triticale. In addition, we aimed to verify the chromosomal locations of these markers using biparental mapping populations and determine their usefulness for breeding purposes in triticale.

## Materials and methods

### Plant material

A range of 232 genetically diverse triticale advanced breeding accessions (193 winter and 39 spring) exhibiting different levels of tolerance to Al was kindly donated for the study by breeders (Małyszyn, Poland).

### Genotypic data

Total genomic DNA was isolated from fresh leaves of 14-day-old seedlings using the Plant DNeasy MiniKit 250 (Qiagen GmbH, Hilden, Germany), following the manufacturer’s instructions. Each triticale accession was represented by a single, randomly selected plant. DNA quantity was measured spectrophotometrically (NanoDrop ND-1000), and its integrity and purity were verified by electrophoresis on 1.2 % agarose gels stained with EtBr (0.1 μg/ml) in TBE.

DNA samples were genotyped at Diversity Arrays Technology Pty Limited (Canberra, Australia; http://www.triticarte.com.au), where DArT markers were evaluated. The putative chromosomal locations of the markers were determined by the DArT P/L company.

### Preliminary analysis of plant materials

Genotypes from triticale accessions were subjected to cluster analysis [UPGMA based on Jaccard’s (Jaccard 1908) genetic distance coefficients]. Computations were performed in PAST Software (Hammer et al. 2001). Similar accessions (at least 95 % similarity) were considered to be identical.

### Phenotypic data

A physiological test for Al tolerance was conducted in hydroponics according to the procedure proposed by Anioł (1984) and described elsewhere (Niedziela et al. 2012, 2014). Triticale accessions were considered tolerant if their average re-growth was ≥5.0 mm. Accessions with re-growth of <2.0 mm and 2.0–5.0 mm were classified as non-tolerant and moderately tolerant, respectively.

### Mapping populations

Mapping of the chromosomal locations of markers was achieved using two biparental F2 triticale mapping populations (*n* = 96 individuals each), namely, MP1 and MP15, derived via crossing individual double haploid plants differing in Al tolerance (Niedziela et al. 2014).

### Identifying DArTs with identical segregation patterns

Forty-nine DArT markers associated with Al tolerance and mapped to chromosomes 4R, 6R, and 7R (Niedziela et al. 2012) were tested for segregation in preselected non-redundant accessions. Markers exhibiting identical or highly similar segregation patterns (95 % similarity based on UPGMA and Jaccard’s coefficient) were considered redundant.

### Identifying redundant DArTs based on their DNA sequences

DArT marker sequences were kindly provided by Dr. A. Kilian (Diversity Arrays Technology P/L, Canberra, Australia), Prof. M. Wędzony (The Franciszek Górski Institute of Plant Physiology of Polish Academy of Sciences, Kraków, Poland), and Dr. hab. M. Tyrka (Rzeszów University of Technology, Department of Biochemistry and Biotechnology, Rzeszów, Poland). The sequences of all chosen DArT markers were >200 bp in length and mapped to chromosomes 4R, 6R, and 7R.

DArT marker sequences were aligned in CLC Main Workbench software version 6.0 (CLC Inc., Aarhus, Denmark; http://www.clcbio.com/). The UPGMA approach was used for clustering. Grouping was verified by bootstrapping with 1000 replicates.

### Conversion of DArT markers to PCR-based assays

#### Primer design

DArT marker DNA sequences were analyzed in CLC Main Workbench software version 6.0 to identify primer pairs for their amplification. The criteria for primer design were as follows: 40–60 % GC rich; minimum annealing temperature, 50 °C; no or negligible secondary structures; and product size ≥80 bp.

#### Specific PCR

Reaction mixtures consisted of 10 ng of total genomic DNA, 50 μmols each of PCR primers, 2.5 mM dNTPs, 2.5 mM MgCl_2_, 1× reaction buffer, and 0.25 U of Taq DNA Polymerase (Qiagen) in a final volume of 10 μl. The thermal profile for each primer pair was tested in gradient thermocycler (PTC-225 Peltier Thermal Cycler; MJ Research). The following profile: [95 °C–7′] [94 °C–10″; X °C–30″; 72 °C–90″] ×45 [72 °C–10′] [5 °C ∞], where X ranged from 48 to 67 °C (Supplementary Table 1) depending on the primer pair, was used. The PCR products were separated on 1.2 % agarose gels in TBE buffer at 5 V/cm for 1 h.

To distinguish DArT markers from their PCR-based counterparts, their names were extended with ‘c’ (i.e., rPt-508078 vs. rPt-508078c).

#### Mapping DArT markers converted to specific PCR assays in F2 populations

Genetic mapping of candidate markers for the Al-tolerance QTL on chromosome 7R (both DArTs and their converted counterparts) was performed in the MP1 and MP15 F2 mapping populations, using the R/qtl package (Broman 2010). All analyses were conducted according to the manual (Broman 2010). If not stated, default options of functions were applied. Duplicated individuals and markers with numerous missing data (>70 %) were omitted using the function subset.cross() and drop.markers(), respectively. Markers with significant distortion (>5 %) were identified using the function geno.table and removed from future analyses. Linkage groups (LGs) were obtained using the logarithm of odds (LOD) set to 15 and a maximum recombination fraction (rf) of 0.35. The best order of markers was determined using the orderMarkers() function with the following parameters: Kosambi mapping function, error probability = 0.005, tolerance for determining convergence = 1e−6, and maximum number of expectation maximization (EM) iterations = 10,000.

#### Marker validation

Converted DArT markers assigned to chromosomes 4R, 6R, and 7R were tested in material from non-redundant triticale breeding accessions. Segregations evaluated based on DArTs (Niedziela et al. 2012) and their converted counterparts were compared for congruency. Spearman rank correlation coefficients between converted markers and Al tolerance of plant materials were calculated using the CORR procedure in SAS 9.3 software (SAS Institute, Cary, NC). Molecular profiles of the converted markers were evaluated and those with the highest Spearman rank correlation with Al-tolerant plants were grouped based on the presence or absence of the marker.

The significance of association between evaluated molecular profiles, including markers with the highest Spearman rank correlations with tolerant and non-tolerant accessions, was assessed via Pearson’s Chi Square test (χ2) (Bewick et al. 2004) using Statistica 12 software (StatSoft 2014). The relative strength of an association between two variables () was calculated according to the following formula,

1 $$\phi = \sqrt {\frac{{\chi_{P}^{2} }}{n}}$$

where *n* is the sample size (Agresti 2002).

## Results

### Analysis of plant materials

An agglomeration approach, performed for 232 triticale breeding accessions using 3117 DArT polymorphic markers, indicated that 161 of the accessions were distinct, including 144 winter and 17 spring genotypes. All non-identical accessions exhibited the same Al tolerance (based on the length of root re-growth) determined in our previous experiments (Niedziela et al. 2012). According to physiological testing, 38 accessions were tolerant to the presence of Al^3+^ ions, 26 were moderately tolerant and 97 were non-tolerant.

### Identifying redundant DArTs based on their segregation and DNA sequences

Agglomeration analyses, based on segregation profiles of 49 DArT markers associated with Al tolerance (Niedziela et al. 2012), showed that 19 were non-redundant in our materials (Supplementary Fig. 1). Of 30 redundant markers, five were assigned to chromosome 4R, eight to two groups (Gr1, Gr2) on chromosome 6R, and seventeen to four groups of redundancy (Gr3, Gr4, Gr5, Gr6) on chromosome 7R (Table 1).

**Table 1:** Arrangement of redundant DArTs based on marker sequence comparison and their segregation pattern

Chromosome	Group	Redundant group on the base of sequence similarity	Redundant groups on the base of segregation
4R	Gr0	rPt-400270; rPt-401376	rPt-400270; rPt-401376; rPt-399885; rPt-390125; rPt-389881
6R	Gr1	rPt-507199; rPt-507896	rPt-507199; rPt-507896; rPt-399834
	Gr2a	rPt-402018; rPt-402447	rPt-402018; rPt-402447; rPt-507674; rPt-399406; rPt-402015
	Gr2b	rPt-507674; rPt-402015	
7R	Gr3a	rPt-508078; rPt-506317; rPt-505798; rPt-509357	rPt-508078; rPt-506317; rPt-505798; rPt-509357; rPt-401366; rPt-509359
	Gr3b	rPt-401366; rPt-509359	
	Gr4a	rPt-505154	rPt-505154; rPt-509056
	Gr4b	rPt-509056	
	Gr5	rPt-401526; rPt-399664	rPt-401526; rPt-399664; rPt-399570; rPt-400816; rPt-399292; rPt-390741; rPt-346936
	Gr6	rPt-401828; rPt-390593	rPt-401828; rPt-390593

A similar analysis, based on 42 available Al-associated DArT marker sequences, revealed the presence of eight clusters encompassing 18 markers, where the sequences of markers within each cluster were almost identical (at least 95 % similarity) (Supplementary Fig. 2; Table 1). One group (Gr0) of markers was assigned to chromosome 4R, three groups (Gr1, Gr2a, Gr2b) were assigned to chromosome 6R and four (Gr3a, Gr3b, Gr5, Gr6) to chromosome 7R (Table 1). The remaining 24 markers have not redundant counterparts.

Evaluation of redundant groups on chromosomes 6R and 7R, identified using segregation data, revealed a number of markers with identical DNA sequences (Table 1). For example, two markers within each of the groups Gr2, Gr3, and G5 had the same sequences (Table 1).

### Conversion of DArTs to specific PCR assays

The available DNA sequences of 42 DArT markers associated with Al tolerance ranged from 106 to 750 nucleotides in length, with two sequences too short for the design of specific primers. Therefore, 40 primers pairs fulfilling the criteria for expected GC content, annealing temperature range, presence of the secondary structures, and length of the amplified fragments were evaluated (Supplementary Table 1); 17 of these primers pairs were found to target redundant markers.

Three primer pairs for DArT marker sequences generated distinct banding profiles from the 161 samples of the association mapping population compared with their DArT counterparts, or resulted in a weak and/or unclear product (Supplementary Table 1). All of these unsatisfactory markers were assigned to chromosome 6R, based on the location of DArT markers according to the DArT P/L company. Three, eight, and thirteen markers from chromosomes 4R, 6R, and 7R, respectively, resulted in polymorphic PCR products of expected sizes and segregated in the same way as their corresponding DArTs. The remaining 13 primers generated monomorphic banding patterns. Hence, although the conversion efficiency of DArTs to specific PCR assays was 100 % (assuming that 40 DArTs could be converted), only 60 % of these proved to be polymorphic among the 161 non-redundant accessions.

### Analysis of the correlation between specific DArT-based PCR markers and Al tolerance

Correlation coefficients between the average value of root regrowth and 24 PCR-based markers associated with Al tolerance were highly significant for 21 of them (Table 2). The highest correlation was observed for rPt-508078c, rPt-401828c and rPt-505154c markers and therefore they were considered for selection purpose. All these markers and their redundant counterparts (Table 2) were assigned to the chromosome 7R.

**Table 2:** Spearman rank correlation coefficient for 161 triticale accessions (144 winter and 17 spring) showing correlation between converted PCR-based markers (‘c’ is added to the original marker code) and average value of root regrowth. Negative values indicate correlation with non-tolerant accessions and positive values with tolerant once

Chromosome	Marker name	Redundant counterparts	Correlation coefficient	Significance *p* value
4R	rPt-507784c	–	−0.239	0.0026
	rPt-505674c	–	0.275	0.0004
	rPt-508577c	–	0.274	0.0004
6R	rPt-399834c	rPt-507199c, rPt-507896c	−0.359	<0.0001
	rPt-401083c	–	0.382	<0.0001
	rPt-505870c	–	0.226	0.0040
	rPt-509167c	–	0.073	0.3566
	rPt-506198c	–	−0.045	0.5908
	rPt-505347c	–	−0.119	0.1300
7R	**rPt-508078c**	**rPt-509357c, rPt-509798c, rPt-506317c, rPt-509359c**	−**0.628**	**<0.0001**
	**rPt-401828c**	**rPt-390593c**	**0.523**	**<0.0001**
	**rPt-505154c**	**rPt-509056c**	−**0.414**	**<0.0001**
	rPt-390741c	rPt-400816c, rPt-401526c,rPt-399664c	−0.307	<0.0001

**Fig. 1:**
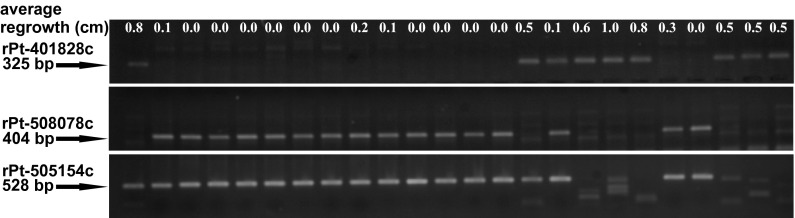
An example of the molecular profiles obtained with rPt-401828c (325 bp), rPt-508078c (404 bp), and rPt-505154c (528 bp) DArT-based specific markers evaluated on agarose gels. The length of root re-growth (in cm) of each plant after Al treatment is indicated at the top of the profile

Marker rPt-401828c was present in 41 of 161 analyzed triticale accessions (Fig. 1). Moreover, this marker was identified almost exclusively among Al-tolerant accessions (33 out of 38 tolerant plants), with only 7 of 97 non-tolerant accessions carrying it. Hence rPt-401828c was highly correlated with Al tolerance (*r* = 0.523, *p* > 0.0001). Another marker, rPt-508078c, was not detected in any tolerant accessions, but was present in 104 non-tolerant and moderately tolerant plants with a correlation to lack of Al tolerance of *r* = 0.628 (*p* > 0.0001). Finally, the marker rPt-505154c was present in 25 tolerant and all non-tolerant and moderately tolerant accessions, equating to the lowest correlation with the trait among the three markers evaluated (*r* = 0.414, *p* > 0.0001).

The Chi square test indicated a significant association (χ2 = 84.539, *p* < 0.0000) between individuals classified into three phenotypic groups (tolerant, moderately tolerant, and non-tolerant) distinguished according to Al response and the molecular profile of the rPt-401828c marker. If two markers were combined, assuming all four dominant profiles (I-0/0, II-0/1, III-1/0, and IV-1/1) generated by markers rPt-508078c and rPt-401828, then the χ2 was 106.715 (*p* < 2.2e−16). Finally, when the third marker, the rPt-505154c, was included in the analysis, the Chi square statistic increased slightly: χ2 = 110.953 (*p* < 2.2e-16). The strengths of the associations (*ϕ*) between one, two, or three marker profiles, and the trait were 0.724, 0.814 and 0.830, respectively.

### Mapping converted DArT markers on chromosome 7R

**Fig. 2:**
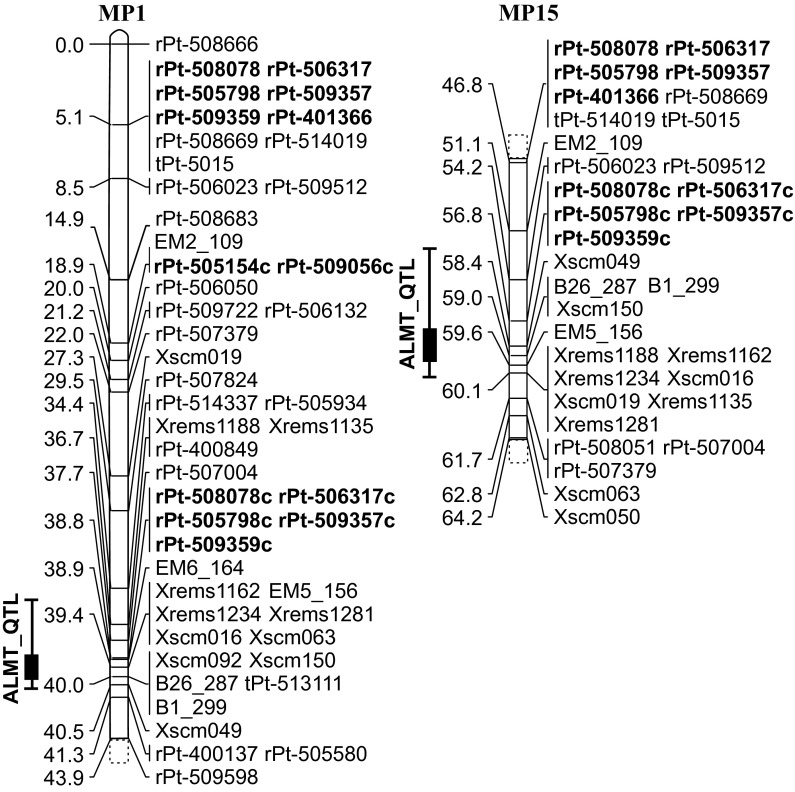
Map of the 7R linkage group evaluated in the MP1 and MP15 F2 mapping populations. The ALMT_QTL is indicated on the left. DArT markers converted to specific PCR assays are shown in red and an additional ‘c’ is added to the end of the original name. Unconverted DArT markers are shown in blue

The converted specific PCR markers (rPt-401828c, rPt-508078c, and rPt-505154c) and their DArTs counterparts associated with Al tolerance on chromosome 7R had similar segregation profiles in an association mapping population (Supplementary Table 1). When tested on F2 biparental mapping populations, MP1 and MP15 (evaluated earlier; Niedziela et al. 2014), only rPt-508078 mapped consistently in both cases. However, it mapped far from the QTL maximum peak at distances of 34.9 and 12.2 cM in MP1 and MP15 populations, respectively. When converted, rPt-508078c mapped very close to the chromosome 7R Al-tolerance QTL maximum, at 1.2 cM (MP1) and 2.2 cM (MP15) (Fig. 2). The rPt-505154 did not map successfully in either population; however, its converted counterpart was found to map 21 cM from the QTL maximum in the MP1 population. The observed differences between DArTs and their converted counterparts were due to the presence of missing data for DArTs and putative differences in allelic parental forms of the two populations.

## Discussion

One of the most important purposes of plant breeding is to prevent decreases in seed yield due to soil acidification, which affects over 50 % of the agricultural land used for plant cultivation (Kochian 1995). Thus, cost-effective and time-efficient methods to distinguish Al-tolerant and non-tolerant triticale plant material are important for the improvement of modern triticale varieties. Until now a physiological test has been used for this purpose (Anioł et al. 1984). While this test is inexpensive, it is based on phenotype, and numerous plants need to be tested to ensure confidence in the results. Therefore, the method of choice is to use DNA markers that are either linked to QTLs or associated with known genes affecting the trait.

To be useful for selection purposes, molecular markers should be inexpensive and adaptable to large scale analyses. Moreover, they need to discriminate between distinct phenotypes with high levels of predictability. Such conditions could be fulfilled by DArT markers, once these are converted into specific PCR assays; however, conversion needs to take account of marker redundancy, at the level of both segregation patterns and DNA sequences. Interestingly, 19 of 49 Al-tolerance associated markers had identical segregation patterns in this study, indicating a high degree of segregation redundancy. Moreover, redundancy was also observed at the level of marker sequences, with 18 of 42 DArT marker sequences forming several groups of redundancy. The presence of redundant markers could be explained by the fact that DArT technology is based on a hybridization approach. The sequences of the probes may not be known, and the same probes may be used under different names. Alternatively, probes having different DNA sequences may be linked together. Thus, the redundancy identified by us is characteristic of DArTs and may reach up to 40 %, as indicated by other investigators (Schouten et al. 2012; Raman et al. 2013). Our study demonstrates that a prescreening step may be useful in significantly limiting the number of markers for conversion or suggesting alternative candidates for conversion.

In our experiments, all DNA probes used for conversion were linked or associated with the trait and had known sequences of the expected size. While we succeeded in converting almost 100 % of markers, only 60 % of these were polymorphic. Clearly, not all primers designed from DArT markers amplify polymorphic fragments (Fiust et al. 2015), likely because the primers do not complement polymorphic sequences. This is a disadvantage of using DArTs to develop PCR assays, since the polymorphisms they identify are generally limited to the restriction sites recognized by endonucleases used in this approach. Moreover, the amplified fragments do not necessarily segregate in the expected manner (Shahin et al. 2009). Thus, determination of chromosomal location and/or association studies are needed to verify whether converted markers are genuinely linked to the trait. Our experimental data from an association mapping population demonstrate that all but three converted and polymorphic markers generated in this study exhibited identical segregation patterns to their DArT counterparts after conversion. It should be stressed however, that the level of DArT marker conversion efficiency is consistent with results presented by others (Fiust et al. 2015).

Analysis of the correlation of converted DArT markers with the trait demonstrated that they may be useful for selection purposes and these data indicate that the marker rPt-401828c could be used independently for selection of tolerant plants. However, incorporation into the analysis of two additional markers associated with lack of Al tolerance (rPt-508078c and rPt-505154c) may increase the stringency of selection and allow the identification of only tolerant plants. Nevertheless, the use of the markers associated with non-tolerant accessions for selection is likely to result in the loss of some tolerant accessions, due to lack of complete linkage between the markers and the trait. It is also important to note that the converted DArTs evaluated by us were dominant markers. Thus, additional experiments will be required to confirm homozygosity of the selected accessions. Finally, in our previous studies (Niedziela et al. 2014) we demonstrated that the strongest Al-tolerance QTL, explaining up to 36 % of the phenotypic variance in triticale, is located on chromosome 7R, indicating that 64 % of the phenotypic variance in this trait is not explained by this QTL. Data consistent with ours were also published for rye (Silva-Navas et al. 2012), providing further evidence that additional factors determine Al tolerance in these plants. A good candidate additional locus that warrants further investigation is the STOP1 transcription factor identified by Silva-Navas et al. (2012) on chromosome 3R. The marker associated with Al tolerance on 3R was also found important in our studies. Unfortunately, the sequence for this marker was not available for use in this study. Further experiments are required to determine the importance of markers associated with STOP1 for MAS purposes.

## Conclusions

Our previous studies of Al tolerance in two triticale biparental mapping populations identified an Al-tolerance QTL on chromosome 7R. Moreover, association mapping demonstrated that the majority of markers associated with the trait were also assigned to this chromosome, with others mapping to chromosomes 3R, 4R, and 6R. The DNA sequences of the majority of DArTs evaluated here were used successfully for the evaluation of converted PCR assays. The converted markers were tested in a large genetic pool of triticale exhibiting distinct reactions to the presence of Al in acidic soils. We selected three markers capable of discriminating tolerant accessions and one of these was mapped to the ALMT QTL maximum for Al tolerance in the MP1 and MP15 biparental populations.

## Electronic supplementary material

Supplementary material 1 (DOCX 21 kb)

Supplementary material 2 (PDF 143 kb)

Supplementary material 3 (DOCX 23 kb)
