# Supplementary material for: Diversity Arrays Technology-based PCR markers for marker assisted selection of aluminum tolerance in triticale (x Triticosecale Wittmack)
Source: Mol Breed. 2015 Nov 3;35(11):209. doi: 10.1007/s11032-015-0400-8 (PMC4631718; doi:10.1007/s11032-015-0400-8)
Supplement: Supplementary file 1 — Supplementary material 1 (DOCX 21 kb) [file 11032_2015_400_MOESM1_ESM.docx]

Article title: Diversity Arrays Technology-based PCR markers for marker assisted selection of aluminum tolerance in triticale (x Triticosecale Wittmack)

Journal name: Molecular Breeding

Author names: Niedziela Agnieszka, Mańkowski Dariusz, Bednarek Piotr T

Affiliation: Plant Breeding and Acclimatization Institute - National Research Institute, Radzików, 05-870 Błonie, Poland

e-mail address of the corresponding author: [p.bednarek@ihar.edu.pl](mailto:p.bednarek@ihar.edu.pl)

Supplementary Figure 1. Clustering DArT markers associated with Al tolerance using Jaccard's genetic distances based on profiles evaluated on 161 triticale breeding accessions. The marker name is identical to that assigned by the DArT PL however, its chromosomal location was added for convenience. Significance of clods was evaluated using 1000 replicates of bootstrapping.
