# Supplementary material for: Diversity Arrays Technology-based PCR markers for marker assisted selection of aluminum tolerance in triticale (x Triticosecale Wittmack)
Source: Mol Breed. 2015 Nov 3;35(11):209. doi: 10.1007/s11032-015-0400-8 (PMC4631718; doi:10.1007/s11032-015-0400-8)

Article title: Diversity Arrays Technology-based PCR markers for marker assisted selection of aluminum tolerance in triticale (x Triticosecale Wittmack)

Journal name: Molecular Breeding

Author names: Niedziela Agnieszka, Mańkowski Dariusz, Bednarek Piotr T

Affiliation: Plant Breeding and Acclimatization Institute - National Research Institute, Radzików, 05-870 Błonie, Poland

e-mail address of the corresponding author: p.bednarek@ihar.edu.pl

Supplementary Figure 2. Clustering of the DArT markers based on a sequence distance method that utilizes the UPGMA algorithm. Values at branches indicate the percent of bootstrap for 1000 replicates. The marker names followed those given on Supplementary Figure 1.

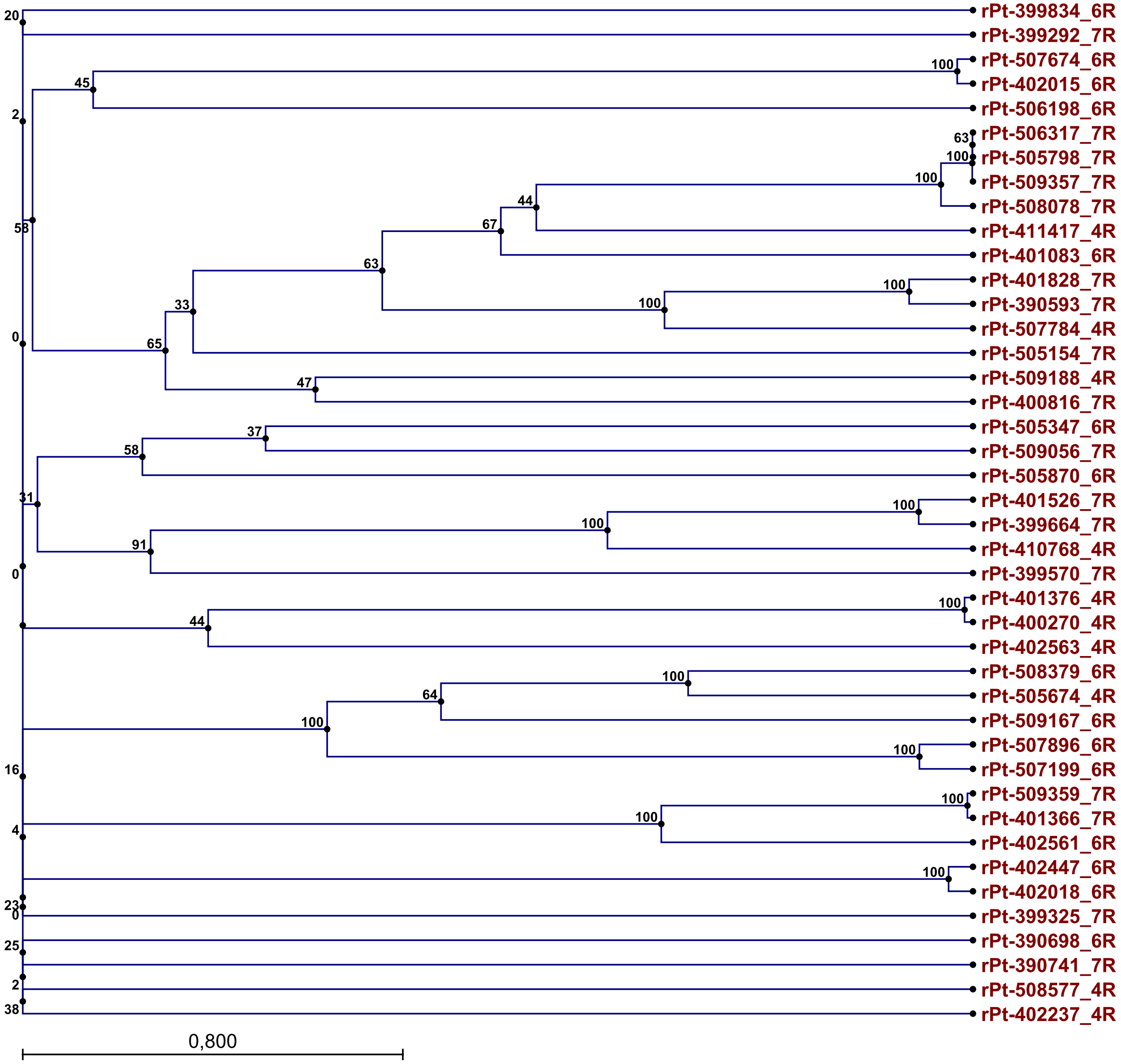

Supplement: Supplementary file 2 — Supplementary material 2 (PDF 143 kb) [file 11032_2015_400_MOESM2_ESM.pdf]
