# Supplementary material for: Diversity Arrays Technology-based PCR markers for marker assisted selection of aluminum tolerance in triticale (x Triticosecale Wittmack)
Source: Mol Breed. 2015 Nov 3;35(11):209. doi: 10.1007/s11032-015-0400-8 (PMC4631718; doi:10.1007/s11032-015-0400-8)
Supplement: Supplementary file 3 — Supplementary material 3 (DOCX 23 kb) [file 11032_2015_400_MOESM3_ESM.docx]

Article title: Diversity Arrays Technology-based PCR markers for marker assisted selection of aluminum tolerance in triticale (x Triticosecale Wittmack)

Journal name: Molecular Breeding

Author names: Niedziela Agnieszka, Mańkowski Dariusz, Bednarek Piotr T

Affiliation: Plant Breeding and Acclimatization Institute - National Research Institute, Radzików, 05-870 Błonie, Poland

e-mail address of the corresponding author: [p.bednarek@ihar.edu.pl](mailto:p.bednarek@ihar.edu.pl)

Supplementary Table 1 The arrangement of data concerning primer pairs designed based on DArT DNA sequences. Only markers amplifying polymorphic signal after conversion were presented in the table.

| No | Chr | Redundant groups | Marker name | Sequence length (bp) | Forward oligonucleotide sequence 5'-->3' | Primer Length (bp) | GC% | Reverse oligonucleotide sequence 3'-->5' | Primer Length (bp) | GC% | Tm Co | Amplified fragment (bp) | Conformity with DArTs segregation |
| --- | --- | --- | --- | --- | --- | --- | --- | --- | --- | --- | --- | --- | --- |
| 1 | 4R | - | rPt-505674 | 691 | AAGTTACTACTGTCAGGC | 18 | 44 | CAACATCAGAAAACCACCA | 19 | 42 | 59.0 | 590 | 100% |
| 2 | 4R | - | rPt-507784 | 621 | AACTACAAACCATAACGGCA | 20 | 40 | CATNTCTTCCACCCGCTC | 18 | 58 | 59.0 | 439 | 100% |
| 3 | 4R | - | rPt-508577 | 411 | GTATAGTTGGTTTCATCGG | 19 | 42 | GGGATAGGATAAATGGAGG | 19 | 47 | 56.0 | 255 | 100% |
| 4 | 6R | Gr1 | rPt-399834 | 274 | CAAGCCATTCTTATCTAACC | 20 | 40 | TCTCTTCAGCTAAGCCTC | 19 | 50 | 54.0 | 170 | 100% |
| 5 | 6R |  | rPt-507199 | 452 | TAGCATTACAATACCCCC | 18 | 44 | ATTTGTNGAGATAGCGAG | 18 | 42 | 54.0 | 228 | 100% |
| 6 | 6R |  | rPt-507896 | 560 | TTACAATACCCCCAAGTC | 18 | 44 | CACCATCTCNTTCTATTCC | 19 | 45 | 54.0 | 428 | 100% |
| 7 | 6R | - | rPt-401083 | 526 | CTTGTATTCGCTTCTGTC | 18 | 44 | TTGTTGTAACTTGGGCTG | 18 | 44 | 56.0 | 465 | 100% |
| 8 | 6R | Gr2 | rPt-402018 | 689 | AGAATGGGACTTGATCAG | 18 | 44 | CCATAAGCAATNTNTGAGAC | 20 | 40 | 52.0 | 582 | 15% |
| 9 | 6R |  | rPt-402447 | 623 | GTGAGTCCACTAGAGCTT | 18 | 50 | CACATCCACCCAACCGTA | 18 | 56 | 56.0 | 504 | 18% |
| 10 | 6R | - | rPt-505870 | 647 | GTTTTTACAGTTTGGCACGA | 20 | 40 | TTGTTTTTGGTGGCGGTGA | 19 | 47 | 54.0 | 536 | 100% |
| 11 | 6R | - | rPt-508379 | 684 | GATATCATGTGCGGCAGG | 18 | 56 | GCGAGTTGACAGTAGTGG | 18 | 56 | 58.0 | 448 | 6% |
| 12 | 6R | - | rPt-509167 | 459 | CTGAGTTTGAGGTTATCC | 18 | 44 | GTAATGAGTAGAGAGCGG | 18 | 50 | 52.0 | 371 | 100% |
| 13 | 6R | - | rPt-506198 | 685 | TCTAGCCAATCCATGCCA | 18 | 50 | CACATCAAAGGCAACACCAA | 20 | 42 | 56.0 | 574 | 100% |
| 14 | 6R | - | rPt-505347 | 530 | ATCCATTCATTCGTCAGC | 18 | 44 | GCATATCTCCATCAAACTCT | 20 | 40 | 56.0 | 446 | 100% |
| 15 | 7R | Gr3 | rPt-508078 | 598 | GCCGAATGACAACAACTTA | 19 | 42 | AGGAGCAGAATGTCAAGAA | 19 | 42 | 59.0 | 404 | 100% |
| 16 | 7R |  | rPt-506317 | 525 | AACCAGATCATTTGCCGA | 18 | 44 | AGCAATGTTAACCCCAAG | 18 | 44 | 48.0 | 482 | 100% |
| 17 | 7R |  | rPt-509357 | 598 | GCCGAATGACAACAACTTA | 19 | 42 | AGGAGCAGAATGTCAAGAA | 19 | 42 | 59.0 | 404 | 100% |
| 18 | 7R |  | rPt-509359 | 525 | ACAGAAGCAACAGCAGGA | 18 | 50 | AGGGAACGCAATGAACAG | 18 | 50 | 59.0 | 240 | 100% |
| 19 | 7R |  | rPt-505798 | 525 | AGGGAACGCAATGAACAG | 18 | 50 | ACAGAAGCAACAGCAGGA | 18 | 50 | 59.0 | 240 | 100% |
| 20 | 7R | Gr4 | rPt-509056 | 552 | GCCCCAATCTGTGTGAAA | 18 | 50 | GCAGATAATGGATTTGGAGT | 20 | 40 | 56.0 | 511 | 100% |
| 21 | 7R |  | rPt-505154 | 547 | CCGCACTTTTTACCAACA | 18 | 44 | ACAGACACAGACCTTTCA | 18 | 44 | 56.0 | 528 | 100% |
| 22 | 7R | Gr5 | rPt-401526 | 618 | TGCTCTCCTCCTCATCGT | 18 | 56 | CGCCCTCTCTTTCTTGAC | 18 | 56 | 52.0 | 556 | 100% |
| 23 | 7R |  | rPt-400816 | 616 | CACCCTCTCTTTCTTGAC | 18 | 50 | CATCCTCTCCTCCTCATC | 18 | 56 | 56.2 | 558 | 100% |
| 24 | 7R |  | rPt-399664 | 642 | AACATCCTCTCCTCCTCATC | 20 | 50 | TCATTTCCTTTCATTGCGCT | 20 | 40 | 58.0 | 306 | 100% |
| 25 | 7R |  | rPt-390741 | 281 | TAACCCTCCGAACAAGACA | 19 | 47 | CNGCGGAGAAAGAGGCAA | 18 | 58 | 56.2 | 169 | 100% |
| 26 | 7R | Gr6 | rPt-390593 | 451 | CCCTTCCAAATTACTCCC | 18 | 50 | TATGCTGCCNTTCTTTCAC | 19 | 45 | 56.0 | 228 | 100% |
| 27 | 7R |  | rPt-401828 | 566 | CCTTCCAAATTACTCCCTC | 19 | 47 | AGATATCATGGGGCCAAA | 18 | 44 | 56.0 | 325 | 100% |
